# Bone Turnover Markers in Adults with Nonalcoholic Fatty Liver Disease: A Systematic Review and Meta-Analysis

**DOI:** 10.1155/2023/9957194

**Published:** 2023-07-19

**Authors:** Chao Li, Yali Cui, Wenjie Zhou, Yiduo Zhang, Xiaocui Huang, Fan Yu

**Affiliations:** ^1^Chengdu Jinjiang District Maternal and Child Healthcare Hospital, Chengdu, China; ^2^Department of Laboratory Medicine, West China Second University Hospital, Sichuan University, Chengdu, China; ^3^Key Laboratory of Birth Defects and Related Diseases of Women and Children (Sichuan University), Ministry of Education, Chengdu, China; ^4^Department of Laboratory Medicine, Meishan Women and Children's Hospital, Alliance Hospital of West China Second University Hospital, Sichuan University, Meishan, China

## Abstract

**Objective:**

Previous studies suggested that the level of bone turnover markers (BTMs) could be altered in patients with nonalcoholic fatty liver disease (NAFLD). We aim to provide a comprehensive understanding on the associations between BTMs and NAFLD in adults with a meta-analysis.

**Methods:**

Articles published up to January 31, 2023, were systematically searched in PubMed, Web of Science, Cochrane database, Embase, and CNKI. The search formula is as follows: “nonalcoholic fatty liver disease” combined with the terms that bone turnover markers such as “osteocalcin,” “collagen type I trimeric cross-linked peptide,” and “procollagen type I N-terminal peptide.” Stata 15.0 software was used to calculate the pooled OR (95% CI) and perform the heterogeneity test, sensitivity analysis, and publication bias.

**Results:**

We identified 18 studies with a total of 12,310 participants. Statistical differences were found between patients with NAFLD compared to the control group for osteocalcin (*n* = 15 studies; SMD: −0.69; 95% CI: −0.73–−0.64; *P*=0.002), procollagen type I N-terminal propeptide (*n* = 5 studies; SMD: −0.40; 95% CI: −0.80−−0.00; *P*=0.049), and collagen type I cross-linked C-telopeptide (*n* = 7 studies; SMD: −0.16; 95% CI: −0.23−−0.09); *P* < 0.001).

**Conclusion:**

Bone turnover markers were lower in patients with NAFLD compared to the control group.

## 1. Introduction

Nonalcoholic fatty liver disease (NAFLD) represents a condition of metabolic stress liver injury with the main characteristics of fat accumulation and hepatic steatosis in liver parenchymal cells, contributing to osteoporosis and cardiovascular disease [1, 2]. As the most prevalent chronic liver disease, NAFLD is not related to a history of excessive alcohol consumption. People around the world are threatened by NAFLD, which is treated as a main factor to affect human health. Its prevalence is as high as 25–30%. The increase in the incidence of NAFLD is mainly caused by changes in daily diet, such as intaking food with high fat and sugar [1, 3]. Patients with NAFLD are associated with osteoporosis, which might be associated with a reduced bone mass [2, 4]. Previous studies have proposed hypotheses such as a systemic inflammatory response in which the liver secretes cytokines when the excess deposition of fatty acids exceeds the compensatory capacity of the liver. This can further alter the bone microenvironment, leading to bone loss and osteoporosis [2, 5]. Studies on osteocalcin showed that bone fat and energy metabolic are affected in patients with NAFLD, with a consequent decrease in osteocalcin levels. The bone fat and energy metabolic are associated with osteocalcin, resulting in osteoporosis [6, 7]. In addition, there are also studies indicating a hepatic origin for some bone turnover markers (BTMs), which has demonstrated that the liver might relate to bone metabolism via multiple pathways [8].

BTMs can reflect bone formation and resorption, the levels of which indicate the current state of bone metabolism. The bone tissues provide structural scaffolding for the body and play an essential role in the metabolic diseases [9]. As a direct marker of bone formation, osteocalcin plays a regulatory role in energy metabolism [10]. Procollagen type I N-terminal propeptide (PINP) and C-terminal cross-linked telopeptide (CTX) are frequently employed in clinical research as BTMs and recommended by the International Osteoporosis Foundation and the International Federation of Clinical Chemistry and Laboratory Medicine [11]. Therefore, the detection of serum BTMs provides an option to dynamically monitor bone formation and bone resorption in a clinical setting.

Mantovani et al.'s meta-analysis showed that nonalcoholic fatty liver disease is associated with a history of osteoporotic fractures but not with low bone mineral density (BMD) [12]. Bone metabolism in patients with NAFLD may have a potential value for screening [13]. However, the association between NAFLD and abnormal bone metabolism as well as BTMs is still unclear. Hence, this systematic review and meta-analysis were conducted to compare BTMs between NAFLD and non-NAFLD subjects, aiming to clarify potential associations between BTM levels and NAFLD.

## 2. Materials and Methods

This systematic review study followed the PRISMA guidelines [14], and the protocol was simultaneously registered in the International Prospective Register of Systematic Reviews (PROSPERO registration number: CRD42022307091).

### 2.1. Search Strategy

We performed a systematic screening via the databases PubMed, Embase, Cochrane Library, Web of Science, and CNKI from the inception to 31 January 2023. The search in PubMed used the following criteria: (osteocalcin OR collagen type I trimeric cross-linked peptide OR collagen type I trimeric cross-linked peptide) AND (nonalcoholic fatty liver disease OR nonalcoholic steatohepatitis) (Supplementary material 1). The search could be traced by citation, and a manual search was also performed to avoid missing relevant publications. Publication retrieval was carried out independently by two researchers, and discrepancies were resolved through consultation with a third researcher.

### 2.2. Selection Criteria

The following paragraphs present the detailed inclusion and exclusion criterion.

#### 2.2.1. Inclusion Criteria

Study subjects were adults aged over 18 years.
(A) NAFLD was diagnosed using liver histology biopsy or imaging methods, such as radiological or ultrasonography diagnosis. (B) Adult participants without NAFLD.
The types of study were cross-sectional, case-control, or cohort design.
The languages were only restricted to English or Chinese.

#### 2.2.2. Exclusion Criteria

1. The study subjects were not performed on humans.
2. The studies were not original (i.e., case reports, commentaries, correspondence, or editorials); the full text of the study was unavailable or the studies provided insufficient data, and the mean or median scores were unable to be calculated.
3. Patients with other disease known to affect bone metabolism were not meta-analyzed, including diabetes mellitus, hyperthyroidism, or rheumatoid arthritis.
4. Additional known causes of chronic hepatic diseases were excluded, including excessive alcohol consumption or hepatitis B virus.
5. Overweight patients accompanied by severe concurrent diseases or patients who were taking medications that affected bone metabolism including those with all kinds of kidney diseases and taking glucocorticoids, with a recent fracture or with cancer.

### 2.3. Data Extraction and Quality Assessment

Data were extracted by two investigators independently, and differences were resolved by a third researcher. The titles, abstracts, and full texts of the relevant studies were extracted and input into a Microsoft Excel datasheet (Supplementary Table S1). The data collected in Excel included authors, years of publication, countries, age of participants, sample size, and percentage of males. The Newcastle–Ottawa Scale (NOS), a recognized tool applicable to the assessment of case-control studies, was employed for study evaluation [15].

### 2.4. Statistical Analysis

All collected data were statistically analyzed using STATA 15.0 (StataCorp, USA), combining standardized mean difference (SMD) with 95% CI. The fixed-effects model or random-effects model was applied according to the degree of heterogeneity, such as *I*^2^ ≤ 50% for the fixed-effects model and *I*^2^ > 50% for the random-effects model. Sensitivity analysis was also performed by excluding each study individually for pooled results with high heterogeneity. *P* values <0.05 were considered as statistical differences. Publication bias was assessed using funnel plots and Egger's test, and subgroup analysis was performed to further identify the sources of heterogeneity by region.

## 3. Results

### 3.1. Search Results Summary

**Figure 1:**
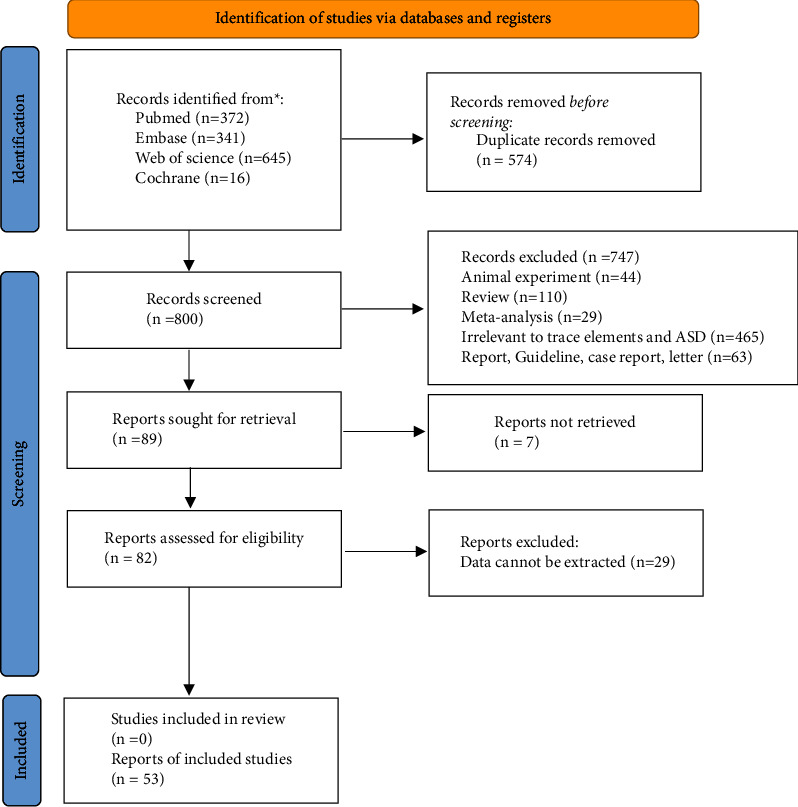
PRISMA flow diagram of the study selection process.

The initial review obtained 322 studies through the five databases (PubMed, *n* = 45; Embase; Cochrane, *n* = 48; Web of Science, *n* = 122; and CNKI, *n* = 57), and 219 studies were left after duplicates removal. By reviewing the titles and abstracts, 171 studies were excluded (22 reviews, eight conference abstracts, 16 randomized controlled trials, four studies on children, and 20 animal studies) and 48 studies with full texts were assessed subsequently. Among them, 21 studies contained incomplete data, seven studies reported other metabolism or diabetes topics, and the full electronic versions of two studies were not available through retrieval.  Figure 1 presents a PRISMA flowchart for the search and study selection.

Finally, 18 studies were included in the systematic review from the references with a total of 12,310 participants. The age ranged from 26 to 88 years, 48.19% being women. The overall NOS score revealed a relatively high quality of the included studies: 16 studies were rated as high quality and the remaining two were median quality (Table 1). Detailed information [16–33] on the included studies (authors, years of publication, regions, sample size, outcomes, and quality assessment score) is shown in Table 1.

### 3.2. Meta-Analyses

**Figure 2:**
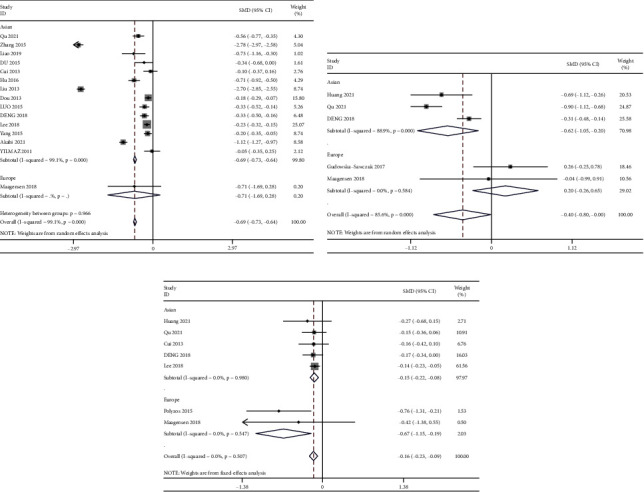
Forest plots of bone turnover markers and nonalcoholic fatty liver disease. (a) Forest plot of osteocalcin and nonalcoholic fatty liver disease. (b) Forest plot of procollagen type I N-terminal propeptide and nonalcoholic fatty liver disease. (c) Forest plot of C-terminal cross-linked telopeptide and nonalcoholic fatty liver disease.

Fifteen studies reported osteocalcin, five included PINP, and seven documented CTX. Three studies measured three of BTMs, while three provided data on two BTMs. Enzyme-linked immunosorbent assay, electrochemiluminescence, and immunoradiometric assay were used to analyze the BTMs. Fifteen studies displayed that patients with NAFLD (*n* = 3479) had a lower osteocalcin level than the control group (SMD = −0.69, 95% CI: −0.73–−0.64, and *P*=0.002; *I*^2^ = 99%) as compared to non-NAFLD individuals (*n* = 8638; Figure 2(a)).

Five studies reported PINP levels among adult NAFLD patients and a control group, displaying a high degree of heterogeneity (*I*^*2*^ = 85.60%). A random-effects model was employed, and the findings indicated lower PINP levels in the NAFLD versus the control group (SMD = −0.40, 95% CI: −0.80–0.00, and *P*=0.049) (Figure 2(b)).

Seven studies evaluated CTX levels among NAFLD patients and the control group, with a nil heterogeneity (*I*^*2*^ = 0.00%) (Figure 2(c)). A fixed-effects model was subsequently adopted to perform the meta-analysis, and the findings indicated lower CTX levels in patients with NAFLD compared to the control group (SMD = −0.16, 95% CI: −0.23–−0.09, and *P* < 0.001).

### 3.3. Subanalyses

Osteocalcin subgroup analysis by regional category showed that Asians individuals with NAFLD had significantly lower osteocalcin levels than the control group (SMD: −0.16; 95% CI: −0.23–−0.09; and *I*^*2*^ = 99.1%), and there were no significant difference in those living in Europe (Figure 2(a)). Subgroup analysis by regional category showed that Asians had a significant decrease in PINP (*I*^*2*^ = 88.9%; SMD: −0.62; and 95% CI: 0.15–−0.20) but not in Europe (*I*^*2*^ = 0.00%; SMD: 0.20; and 95% CI: −0.26–−0.65]) (Figure 2(b)).

### 3.4. Sensitivity Analyses and Publication Bias

**Figure 3:**
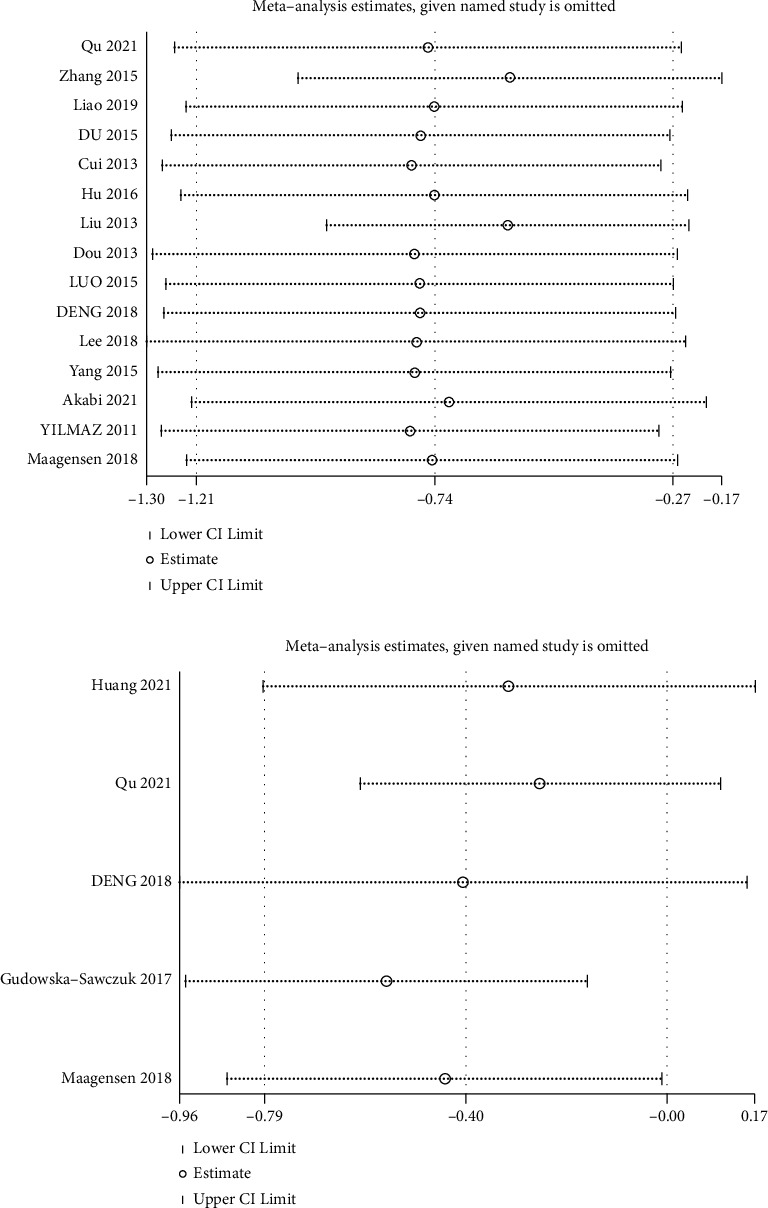
Sensitivity analysis of the studied bone turnover markers and nonalcoholic fatty liver disease. (a) Osteocalcin and nonalcoholic fatty liver disease. (b) Sensitivity analysis of procollagen type I N-terminal propeptide and nonalcoholic fatty liver disease.

**Figure 4:**
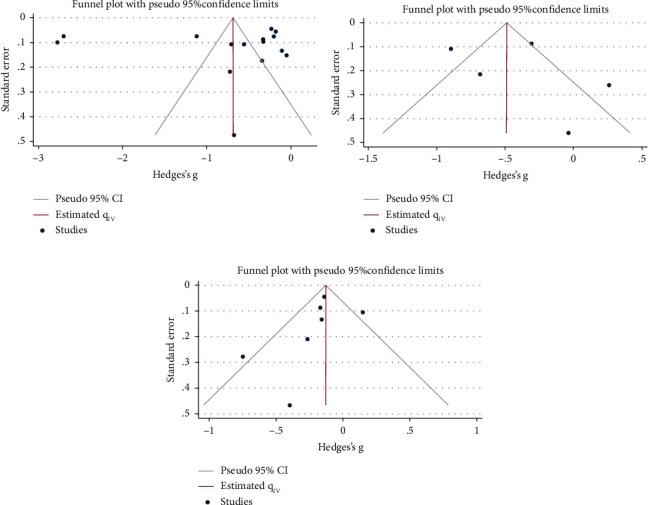
Funnel plots for the risk of bone turnover markers and nonalcoholic fatty liver disease. (a) Funnel plots osteocalcin and nonalcoholic fatty liver disease. (b) Funnel plots of procollagen type I N-terminal propeptide and nonalcoholic fatty liver disease. (c) Funnel plots of C-terminal cross-linked telopeptide and nonalcoholic fatty liver disease.

There were high degrees of heterogeneity for osteocalcin (*I*^*2*^ = 99%) and PINP (*I*^*2*^ = 85.6%). The detailed results of the sensitivity analyses are shown Figure 3. None of the three studies affected the results (Figure 3). Funnel plots demonstrated no asymmetry (Figure 4), and Egger's tests revealed no publication bias among the included studies.

## 4. Discussion

NAFLD has been recognized as a risk factor for osteoporosis. Our systematic review and meta-analysis demonstrated that osteocalcin, procollagen type I N-terminal propeptide, and collagen type I cross-linked C-telopeptide in patients with NAFLD have significantly lower levels than the control group. The results of subgroup analysis suggested that the region could be the sources of heterogeneity in this meta-analysis. Funnel plots and Egger's tests revealed no publication bias among the included studies. Sensitivity analyses showed no meaningful differences including these studies. None of these findings affected the results of the meta-analysis and the general trend towards a reduction in BTMs in NAFLD.

The approximate prevalence of NAFLD is 25.24% worldwide [34]. Mantovani et al. [12] reported that NAFLD is related to osteoporotic fractures rather than BMD. Recently, a study showed that there is a significant detrimental effect of NAFLD on BMD [35]. It seems that there are inconsistent findings regarding of the associations between NAFLD and BMD. Theoretically, people with NAFLD usually intend to gain weight and body mass index due to metabolic disorders and then bone loading might be increased subsequently. Bone turnover biomarkers (e.g., osteocalcin, procollagen type I N-terminal propeptide, and collagen type I cross-linked C-telopeptide) have been measured in individuals with NAFLD, but none of these bone biomarkers have been shown to correlate with NAFLD in meta-analysis. Our study first showed a significant pattern that bone turnover makers decreased with NAFLD at the individual level. Although we conducted the subgroup analysis among a region, data from more regions should be considered in the further studies to provide further insights into it. Due to differences in ethnicity, geography, and lifestyle, bone metabolism also varies between regions [36].

The present results have shown the important clinical implication that the decreasing of BTMs could be one of the risk factors for the development of osteoporosis in adults with NAFLD [37]. It is crucial to understand these changes in BTMs because BTMs have been reported to be related with an increased risk of osteoporosis and fracture in NAFLD. The previous studies have shown that BMD was a crucial factor in assessing the severity of osteoporosis and it helps to accurately forecast the risk of fractures [38, 39]. Compared to BMD, bone turnover markers can timely and accurately reflect the state of bone metabolism of the patients. Some studies have shown that PINP and serum 25-hydroxyvitamin D3 are important reference indicators for the diagnosis and treatment of osteoporosis [40, 41]. Changes in PINP in early treatment are correlated with changes in lumbar BMD at 18 months of treatment [42]. Vitamin D levels are greatly reduced in NAFLD patients, and liver steatosis can be reversed after vitamin D supplementation [43]. Liu et al. have shown that serum 25-hydroxyvitamin D3 and PINP are decreased in NAFLD patients and serum PINP is positively correlated to serum 25-hydroxyvitamin D3 [44]. Furthermore, patients with moderate or severe NAFLD had an increased risk of developing osteoporosis [37] and an increased fracture risk occurrence as the lower value of bone turnover markers [45, 46].

It had been reported that reduced physical activity, insulin resistance, growth hormone/insulin-like growth factor (GH/IGF-1) axis, vitamin D3 deficiency, and chronic inflammation have been recognized as mediators of mutual interactions between the skeleton and the liver. The GH/IGF-1 axis is involved in skeletal muscle protein metabolism, bone growth, and remodeling [2, 47]. In addition, bone fat and energy metabolism are associated with serum osteocalcin which is a good predictor of the severity of hepatic steatosis with a sensitivity and specificity of 80% when its level is less than 44.5 ng/mL [48, 49]. Therefore, the serum screening of BTMs is a key strategy and possible theoretical basis for detecting osteoporosis in the NAFLD population.

### 4.1. Limitations and Strength

Admittedly, there are several limitations in our study. First, only observational studies were meta-analyzed and it might be too early to draw definitive conclusions as other factors may underpin the observed results. Another disadvantage is that although we thoroughly searched for published studies, the included studies are mostly adults from Asia rather than other populations, which may explain the potential publication bias of BTMs, as Asian and non-Asian populations have differences in adipose tissue composition, diet, and genetic and cultural backgrounds which may adversely affect the risk of both osteoporosis and osteoporotic fractures. Thus, it does highlight the need for larger studies. However, our study provided a comprehensive understanding on BTMs and NAFLD. The results of this meta-analysis are robust according to the sensitivity analyses.

## 5. Conclusion

The results of this systematic review and meta-analysis suggest that BTMs are lower in patients with NAFLD compared to those without the disease. Prospective studies, particularly from other world regions, and mechanistic studies are needed to gain an understanding of the relationship between NAFLD and osteoporosis and to fix the role in clinical practice.

## Figures and Tables

**Table 1:** Characteristics of the included studies in the meta-analysis.

Nos.	First authors	Years	Region	Population	Age (mean, SD)	Gender (male, female)	Outcomes	Quality assessment
1	Huang [16]	2021	China	90	38.86 ± 6.83	46, 44	PINP CTX	7
2	Qu [17]	2021	China	360	55.65 ± 9.13	191, 169	PINP CTX OC	7
3	Zhang [18]	2015	China	800	54.10 ± 4.47	0, 800	OC	7
4	Xin [19]	2019	China	88	40.88 ± 5.54	38, 50	OC	7
5	Du [20]	2015	China	174	66.89 ± 10.18	123, 51	OC	6
6	Cui [21]	2013	China	224	59.55 ± 5.73	99, 125	OC CTX	7
7	Hu [22]	2016	China	368	40.11 ± 10.18	273, 95	OC	7
8	Liu [23]	2013	China	1683	37.52 ± 11.23	1683, 0	OC	7
9	Dou [24]	2013	China	1558	54.02 ± 8.67	1558, 0	OC	8
10	Luo [25]	2015	China	733	56.24 ± 4.51	0, 733	OC	7
11	Deng [26]	2018	China	540	50.42 ± 5.05	540, 0	PINP CTX OC	6
12	Gudowska-Sawczuk [27]	2017	Poland	58	26–88	30, 28	PINP	5
13	Lee [28]	2018	Korea	3737	54.48 ± 2.50	0, 3737	CTX, OC	6
14	Yang [29]	2015	Korea	859	45.00 ± 6.72	859, 0	OC	8
15	Al-Akabi [30]	2021	Iraq	800	30–50	800, 0	OC	5
16	Yilmaz [31]	2011	Turkey	174	48 ± 7.56	87, 87	OC	7
17	Polyzos [32]	2015	Japan	47	53.03 ± 2.46	34, 13	CTX	6
18	Maagensen [33]	2018	Denmark	17	52.97 ± 16.93	17, 0	PINP CTX OC	6

PINP, procollagen type I N-terminal propeptide; CTX, C-terminal cross-linked telopeptide; OC, osteocalcin.

## Data Availability

The original contributions presented in the study are included in the article/supplementary material. Further inquiries can be directed to the corresponding author.

## Supplementary Materials

Supplementary Materials Supplementary Material 1: search strategy. Supplementary Table S1.
