# Corrigendum to “Bone Turnover Markers in Adults with Nonalcoholic Fatty Liver Disease: A Systematic Review and Meta-Analysis”

**DOI:** 10.1155/ije/9836309

**Published:** 2025-08-15

C. Li, Y. Cui, W. Zhou, Y. Zhang, X. Huang, and F. Yu, “Bone Turnover Markers in Adults with Nonalcoholic Fatty Liver Disease: A Systematic Review and Meta-Analysis,” *International Journal of Endocrinology* 2023 (2023): 9957194, https://doi.org/10.1155/2023/9957194.

In the article titled “Bone Turnover Markers in Adults with Nonalcoholic Fatty Liver Disease: A Systematic Review and Meta-Analysis,” there was an error in Figure 1. The corrected figure is shown below.

We apologize for this error.

## Figures and Tables

**Figure 1:**
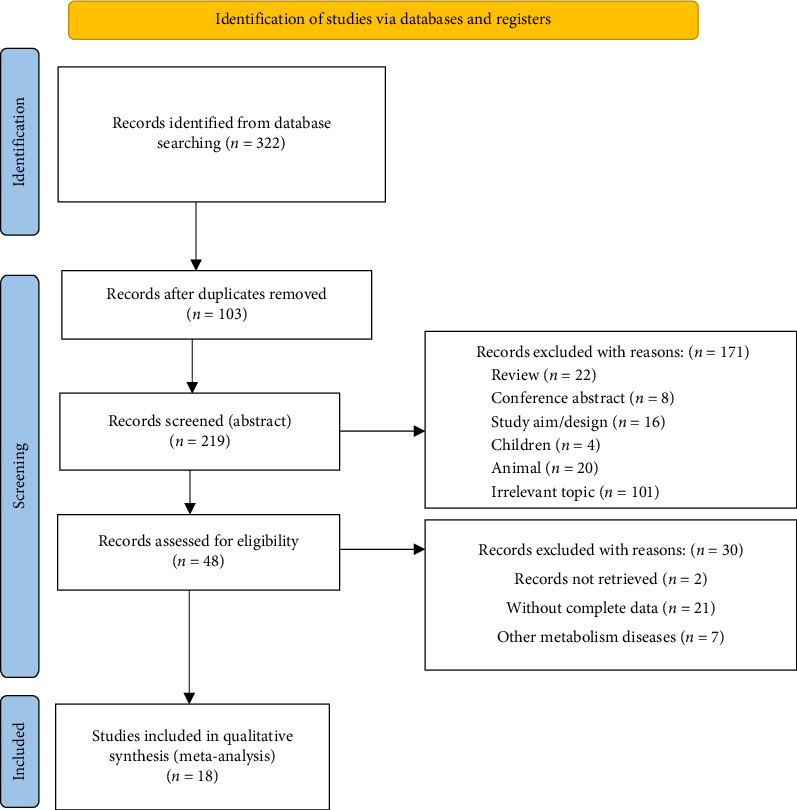
PRISMA flow diagram of the study selection process. PRISMA 2020 flow diagram for new systematic reviews which included searches of databases and registers only.

