# Supplementary material for: Bone Turnover Markers in Adults with Nonalcoholic Fatty Liver Disease: A Systematic Review and Meta-Analysis
Source: Int J Endocrinol. 2023 Jul 19;2023:9957194. doi: 10.1155/2023/9957194 (PMC11390221; doi:10.1155/2023/9957194)
Supplement: Supplementary Materials — Supplementary Material 1: search strategy. Supplementary Table S1. [file 9957194.f1.docx]

**Supplementary Data S1** Search strategy

| Database | PubMed, Embase, Cochrane Library, Web of science, CNKI | | | | | | | |
| --- | --- | --- | --- | --- | --- | --- | --- | --- |
| PICO | P: no limitation  E:nonalcoholic fatty liver disease(NAFLD)  C: people that are not NAFLD  O: serum concentration of bone turnover markers ：Osteocalcin、β-CTX、 P1NP  S: observational studies including cohort study, case control study or cross sectional .  Question: | | | | | | | |
| Search& bibliograghy |  | | | | | | | |
|  |  | Database | Search formula “Osteocalcin” [MeSH] | | | | Items |  |
|  |  | PubMed | \| #1 \| “Osteocalcin” [MeSH] OR “Osteocalcin” OR “Bone gamma-Carboxyglutamic Acid Protein” OR “Bone gamma Carboxyglutamic Acid Protein” OR “Vitamin K- Dependent Bone Protein” OR “Vitamin K Dependent Bone Protein” OR “Calcium- Binding Protein, Vitamin K- Dependent” OR “Gla Protein, Bone” OR “4-Carboxyglutamic Protein, Bone” OR “Bone 4-Carboxyglutamic Protein” OR “Protein, Bone 4-Carboxyglutamic” OR “Bone Gla Protein” OR “4 carboxyglutamic acid containing protein, bone” OR “4 carboxyglutamic acid protein” OR “bone 4 carboxyglutamic acid containing protein” OR “bone gamma carboxyglutamic acid containing protein” OR “bone gla containing protein” OR “bone Gla protein” OR “Bone 4-Carboxyglutamic Protein” OR “Bone gamma-Carboxyglutamic Acid Protein” OR “Gla Protein, Bone” OR “Calcium Binding Protein, Vitamin K Dependent” OR “Bone gamma Carboxyglutamic Acid Protein” OR “Vitamin K- Dependent Bone Protein” OR “Protein, Bone Gla” OR “4 Carboxyglutamic Protein, Bone” OR “Vitamin K Dependent Bone Protein” \| \| --- \| --- \| \| #2 \| “collagen type I trimeric cross-linked peptide” [MeSH] OR “collagen type I trimeric cross-linked peptide” OR ”Bone gamma- Carboxyglutamic Acid Protein” OR ”trimeric cross-linked peptide collagen type I” OR ”C-terminal type I collagen telopeptide” OR ”CTCLP” OR ”CTx telopeptide” OR ”serum carboxyterminal telopeptide type I collagen” OR ”ICTP peptide” OR ”C-terminal telopeptide of type I collagen” OR ”COOH-terminal telopeptide of type I collagen” OR ”C-terminal cross- linking telopeptide, collagen type I” OR ”N-telopeptide” OR ”N-terminal type I collagen telopeptide” OR ”NTx telopeptide” OR ”pyridinoline cross-linked carboxy-terminal telopeptide, collagen type I” OR ”C-telopeptide” OR ”i- ICTP” OR ”CTX” \| \| #3 \| “procollagen Type I N-terminal peptide” [MeSH] OR “procollagen Type I N-terminal peptide” OR “P- I-P peptide” OR “Type I procollagen N-terminal peptide” OR “P1NP peptide” OR “amino terminal propeptide of type I collagen” OR “PINP peptide” OR “fetal antigen 2, human” OR “foetal antigen 2, human” OR “FA2 antigen, human” OR “aminoterminal propeptide of type I collagen, human” OR “collagen type I, amino-propeptide, human” OR ”PINP” \| \| #4 \| “Non alcoholic Fatty Liver Disease” [MeSH] OR “NAFLD” OR ”Non alcoholic Fatty Liver Disease” OR ”Nonalcoholic Fatty Liver Disease” OR ”Fatty Liver, Nonalcoholic” OR ”Fatty Livers, Nonalcoholic” OR ”Liver, Nonalcoholic Fatty” OR ”Livers, Nonalcoholic Fatty” OR ”Nonalcoholic Fatty Liver” OR ”Nonalcoholic Fatty Livers” OR ”Nonalcoholic Steatohepatitis” OR ” Nonalcoholic Steatohepatitides” OR ”Steatohepatitides, Nonalcoholic” OR ”hepato-steatosis” OR ”non alcoholic hepatosteatosis” OR ”non alcoholic liver steatosis” OR “non alcoholic steatotic hepatopathy” OR “non-alcoholic fatty liver” OR “non-alcoholic fatty liver disease” OR “non-alcoholic FLD” OR “non-alcoholic hepatic steatosis” OR ”nonalcoholic FLD” OR “nonalcoholic hepatosteatosis” OR ”nonalcoholic hepatic steatosis” OR ”nonalcoholic liver steatosis” OR ” Steatohepatitis, Nonalcoholic” OR “Steatohepatitides, Nonalcoholic” OR “Nonalcoholic Steatohepatitis” OR “Nonalcoholic Steatohepatitides” OR “Fatty Livers, Nonalcoholic” OR “Liver, Nonalcoholic Fatty” OR “Nonalcoholic Fatty Livers” OR “Livers, Nonalcoholic Fatty” OR “NAFLD” OR “Non alcoholic Fatty Liver Disease” OR “Nonalcoholic Fatty Liver” OR “Nonalcoholic Fatty Liver Disease” OR “Fatty Liver, Nonalcoholic” \| \| #5 \| “nonalcoholic steatohepatitis” [MeSH] OR “NASH” OR ”nonalcoholic steatohepatitis” OR ”non alcohol steato-hepatitis” OR ” non alcohol steatohepatitis” OR ”non alcoholic steato-hepatitis” OR ”non-alcohol steato-hepatitis” OR ”non-alcohol steatohepatitis” OR ”non-alcoholic steatohepatitis” OR ”non-alcoholic steatosis hepatitis” OR ”non-alcoholic steatotic hepatitis” OR ”nonalcohol steato-hepatitis” OR ”nonalcohol steatohepatitis” OR ”nonalcoholic fatty liver inflammation” OR ”nonalcoholic steato-hepatitis “ OR ”nonalcoholic steatosis hepatitis” OR ”nonalcoholic steatotic hepatitis” \| \| #6 \| (#5 or #4) and (#1 or #2 or #3) \| | | | | 20618  17197  1564  32915  44070  45 |  |
|  |  | Embase |  | | | | 35352  25204  3226  36664  63558  50 |  |
|  |  |  |  | #1 | 'osteocalcin'/exp OR 'osteocalcin' OR 'protein, bone 4 carboxyglutamic' OR '4 carboxyglutamic acid containing protein, bone' OR '4 carboxyglutamic acid protein' OR 'bone 4 carboxyglutamic acid containing protein' OR 'bone gamma carboxyglutamic acid containing protein' OR 'bone gla containing protein' OR 'bone gla protein' OR 'bone 4 carboxyglutamic protein' OR 'gla protein, bone' OR 'calcium binding protein, vitamin k dependent' OR 'protein, bone gla' OR '4 carboxyglutamic protein, bone' OR 'vitamin k dependent bone protein' |  |  |  |
|  |  |  |  | #2 | 'collagen type i trimeric cross linked peptide'/exp OR 'collagen type i trimeric cross linked peptide' OR 'bone gamma carboxyglutamic acid protein' OR 'trimeric cross linked peptide collagen type i' OR 'c terminal type i collagen telopeptide' OR 'ctclp' OR 'ctx telopeptide' OR 'serum carboxyterminal telopeptide type i collagen' OR 'ictp peptide' OR 'c terminal telopeptide of type i collagen' OR 'cooh terminal telopeptide of type i collagen' OR 'c terminal cross linking telopeptide, collagen type i' OR 'n telopeptide' OR 'n terminal type i collagen telopeptide' OR 'ntx telopeptide' OR 'pyridinoline cross linked carboxy terminal telopeptide, collagen type i' OR 'c telopeptide' OR 'i ictp' OR 'ctx' |  |  |  |
|  |  |  |  | #3 | 'procollagen type i n terminal peptide'/exp OR 'procollagen type i n terminal peptide' OR 'p i p peptide' OR 'type i procollagen n terminal peptide' OR 'p1np peptide' OR 'amino terminal propeptide of type i collagen' OR 'pinp peptide' OR 'fetal antigen 2, human' OR 'foetal antigen 2, human' OR 'fa2 antigen, human' OR 'aminoterminal propeptide of type i collagen, human' OR 'collagen type i, amino propeptide, human' OR 'pinp' |  |  |  |
|  |  |  |  | #4 | 'nonalcoholic steatohepatitis'/exp OR 'nash' OR 'non alcoholic steato hepatitis' OR 'non alcohol steato hepatitis' OR 'non alcohol steatohepatitis' OR 'non alcoholic steatohepatitis' OR 'non alcoholic steatosis hepatitis' OR 'non alcoholic steatotic hepatitis' OR 'nonalcohol steato hepatitis' OR 'nonalcohol steatohepatitis' OR 'nonalcoholic fatty liver inflammation' OR 'nonalcoholic steato hepatitis' OR 'nonalcoholic steatosis hepatitis' OR 'nonalcoholic steatotic hepatitis' |  |  |  |
|  |  |  |  | #5 | “ nonalcoholic steatohepatitis ” [MeSH] OR “NASH” OR ”nonalcoholic steatohepatitis ” OR ”non alcohol steato-hepatitis ” OR ” non alcohol steatohepatitis ” OR ”non alcoholic steato-hepatitis ” OR ”non-alcohol steato-hepatitis ” OR ”non-alcohol steatohepatitis ” OR ”non-alcoholic steatohepatitis ” OR ”non-alcoholic steatosis hepatitis ” OR ”non-alcoholic steatotic hepatitis ” OR ”nonalcohol steato-hepatitis ” OR ”nonalcohol steatohepatitis ” OR ”nonalcoholic fatty liver inflammation” OR ” nonalcoholic steato-hepatitis “ OR ”nonalcoholic steatosis hepatitis ” OR ”nonalcoholic steatotic hepatitis ” |  |  |  |
|  |  |  |  | #6 | (#5 or #4) and (#1 or #2 or #3) AND 'article'/it |  |  |  |
|  |  |  |  | | | |  |  |

| Cochrane Library | #1  #2  #3  #4  #5  #6 | | ‘Osteocalcin’ OR ‘Bone gamma Carboxyglutamic Acid Protein’ OR ‘Bone gamma Carboxyglutamic Acid Protein’ OR ‘Vitamin K Dependent Bone Protein’ OR ‘Vitamin K Dependent Bone Protein’ OR ‘Calcium Binding Protein, Vitamin K Dependent’ OR  ‘Gla Protein, Bone’ OR ‘4 Carboxyglutamic Protein, Bone’ OR ‘Bone 4 Carboxyglutamic Protein’ OR ‘Protein, Bone 4 Carboxyglutamic’ OR ‘Bone Gla Protein’ OR ‘4 carboxyglutamic acid containing protein, bone ’ OR ‘4 carboxyglutamic acid protein ’ OR ‘bone 4 carboxyglutamic acid containing protein ’ OR ‘bone gamma carboxyglutamic acid containing protein ’ OR ‘ bone gla containing protein ’ OR ‘ bone Gla protein ’ OR ‘Bone 4 Carboxyglutamic Protein’ OR ‘Bone gamma Carboxyglutamic Acid Protein’ OR ‘Gla Protein, Bone’ OR ‘Calcium Binding Protein, Vitamin K Dependent’ OR ‘Bone gamma Carboxyglutamic  Acid Protein’ OR ‘Vitamin K Dependent Bone Protein’ OR ‘Protein, Bone Gla’ OR ‘4 Carboxyglutamic Protein, Bone’ OR ‘Vitamin K Dependent Bone Protein’ |  | 2548  3656  4729  3951  2992  48 |  |
| --- | --- | --- | --- | --- | --- | --- |
|  |  |  | ‘collagen type I trimeric cross linked peptide ’ OR ‘Bone gamma Carboxyglutamic Acid Protein’ OR ‘trimeric cross linked peptide collagen type I’ OR ‘C terminal type I collagen telopeptide ’ OR ‘CTCLP’ OR ‘CTx telopeptide ’ OR ‘serum carboxyterminal telopeptide type I collagen ’ OR ‘ICTP peptide ’ OR ‘C terminal telopeptide of type I collagen ’ OR ‘COOH terminal telopeptide of type I collagen ’ OR ‘C terminal cross linking telopeptide, collagen type I’ OR ‘N telopeptide ’ OR ‘N terminal type I collagen telopeptide’ OR ‘NTx telopeptide ’ OR ‘ pyridinoline cross linked carboxy terminal telopeptide, collagen type I’ OR ‘C telopeptide ’ OR ‘i ICTP’ OR ‘CTX’ |  |  |  |
|  |  |  | ‘ procollagen Type I N terminal peptide ’ OR ‘P I P peptide ’ OR ‘Type I procollagen N terminal peptide ’ OR ‘P1NP peptide ’ OR ‘amino terminal propeptide of type I collagen ’ OR ‘PINP peptide ’ OR ‘fetal antigen 2, human ’ OR ‘foetal antigen 2, human ’ OR ‘FA2 antigen, human ’ OR ‘aminoterminal propeptide of type I collagen, human’ OR ‘collagen type I, amino propeptide, human ’ OR ‘PINP’ |  |  |  |
|  |  |  | ‘NAFLD’ OR ‘Non alcoholic Fatty Liver Disease’ OR ‘Nonalcoholic Fatty Liver Disease’ OR ‘Fatty Liver, Nonalcoholic’ OR ‘Fatty Livers, Nonalcoholic’ OR ‘Liver, Nonalcoholic Fatty’ OR ‘Livers, Nonalcoholic Fatty’ OR ‘Nonalcoholic Fatty Liver’ OR  ‘Nonalcoholic Fatty Livers’ OR ‘Nonalcoholic Steatohepatitis’ OR ‘Nonalcoholic Steatohepatitides’ OR ‘Steatohepatitides, Nonalcoholic’ ‘OR ‘hepato steatosis’ OR ‘non alcoholic hepatosteatosis ’ OR ‘non alcoholic liver steatosis ’ OR ‘non alcoholic steatotic hepatopathy ’ OR ‘non alcoholic fatty liver ’ OR ‘non alcoholic fatty liver disease ’ OR ‘non alcoholic FLD’ OR ‘non alcoholic hepatic steatosis ’ OR ‘‘OR ‘nonalcoholic FLD’ OR ‘nonalcoholic hepatosteatosis ’ OR ‘nonalcoholic hepatic steatosis ’ OR ‘ nonalcoholic liver steatosis ’ OR ‘Steatohepatitis, Nonalcoholic’ OR ‘Steatohepatitides, Nonalcoholic’ OR ‘Nonalcoholic Steatohepatitis’ OR ‘Nonalcoholic Steatohepatitides’ OR ‘Fatty Livers, Nonalcoholic’ OR ‘Liver, Nonalcoholic Fatty’ OR ‘Nonalcoholic Fatty Livers’ OR ‘Livers, Nonalcoholic Fatty’ OR ‘NAFLD’ OR ‘Non alcoholic Fatty Liver Disease’ OR ‘Nonalcoholic Fatty Liver’ OR ‘Nonalcoholic Fatty Liver Disease’ OR ‘Fatty Liver, Nonalcoholic’ |  |  |  |
|  |  |  | ‘NASH’ OR ‘ nonalcoholic steatohepatitis ’ OR ‘non alcohol steato hepatitis ’ OR ‘non alcohol steatohepatitis ’ OR ‘non alcoholic steato hepatitis ’ OR ‘non alcohol steato hepatitis ’ OR ‘non alcohol steatohepatitis ’ OR ‘non alcoholic steatohepatitis ’ OR ‘non alcoholic steatosis hepatitis ’ OR ‘non alcoholic steatotic hepatitis ’ OR ‘nonalcohol steato hepatitis ’ OR ‘nonalcohol steatohepatitis ’ OR ‘nonalcoholic fatty liver inflammation ’ OR ‘nonalcoholic steato hepatitis ‘ OR ‘nonalcoholic steatosis hepatitis ’ OR ‘nonalcoholic steatotic hepatitis ’ |  |  |  |
|  |  |  | (#5 or #4) and (#1 or #2 or #3) |  |  |  |
| Web of  science |  | | | | 25445  25430 |  |
|  |  | #1 | TI=((Osteocalcin) OR (Bone gamma Carboxyglutamic Acid Protein) OR (Bone gamma Carboxyglutamic Acid Protein) OR (Vitamin K Dependent Bone Protein) OR (Vitamin K Dependent Bone Protein) OR (Calcium Binding Protein, Vitamin K Dependent) OR (Gla Protein, Bone) OR (4 Carboxyglutamic Protein, Bone) OR (Bone 4 Carboxyglutamic Protein) OR (Protein, Bone 4 Carboxyglutamic) OR (Bone Gla Protein) OR (4 carboxyglutamic acid containing protein, bone) OR (4 carboxyglutamic acid protein) OR (bone 4 carboxyglutamic acid containing protein) OR (bone gamma carboxyglutamic acid containing protein) OR (bone gla containing protein) OR (bone Gla protein) OR (Bone 4 Carboxyglutamic Protein) OR (Bone gamma Carboxyglutamic Acid Protein) OR (Gla Protein, Bone) OR (Calcium Binding Protein, Vitamin K Dependent) OR (Bone gamma Carboxyglutamic Acid Protein) OR (Vitamin K Dependent Bone Protein) OR (Protein, Bone Gla) OR (4 Carboxyglutamic Protein, Bone) OR (Vitamin K Dependent Bone Protein)) OR AB=((Osteocalcin) OR (Bone gamma Carboxyglutamic Acid Protein) OR (Bone gamma Carboxyglutamic Acid Protein) OR (Vitamin K Dependent Bone Protein) OR (Vitamin K Dependent Bone Protein) OR (Calcium Binding Protein, Vitamin K Dependent) OR (Gla Protein, Bone) OR (4 Carboxyglutamic Protein, Bone) OR (Bone 4 Carboxyglutamic Protein) OR (Protein, Bone 4 Carboxyglutamic) OR (Bone Gla Protein) OR (4 carboxyglutamic acid containing protein, bone) OR (4 carboxyglutamic acid protein) OR (bone 4 carboxyglutamic acid containing protein) OR (bone gamma carboxyglutamic acid containing protein) OR (bone gla containing protein) OR (bone Gla protein) OR (Bone 4 Carboxyglutamic Protein) OR (Bone gamma Carboxyglutamic Acid Protein) OR (Gla Protein, Bone) OR (Calcium Binding Protein, Vitamin K Dependent) OR (Bone gamma Carboxyglutamic Acid Protein) OR (Vitamin K Dependent Bone Protein) OR (Protein, Bone Gla) OR (4 Carboxyglutamic Protein, Bone) OR (Vitamin K Dependent Bone Protein)) OR AK=((Osteocalcin) OR (Bone gamma Carboxyglutamic Acid Protein) OR (Bone gamma Carboxyglutamic Acid Protein) OR (Vitamin K Dependent Bone Protein) OR (Vitamin K Dependent Bone Protein) OR (Calcium Binding Protein, Vitamin K Dependent) OR (Gla Protein, Bone) OR (4 Carboxyglutamic Protein, Bone) OR (Bone 4 Carboxyglutamic Protein) OR (Protein, Bone 4 Carboxyglutamic) OR (Bone Gla Protein) OR (4 carboxyglutamic acid containing protein, bone) OR (4 carboxyglutamic acid protein) OR (bone 4 carboxyglutamic acid containing protein) OR (bone gamma carboxyglutamic acid containing protein) OR (bone gla containing protein) OR (bone Gla protein) OR (Bone 4 Carboxyglutamic Protein) OR (Bone gamma Carboxyglutamic Acid Protein) OR (Gla Protein, Bone) OR (Calcium Binding Protein, Vitamin K Dependent) OR (Bone gamma Carboxyglutamic Acid Protein) OR (Vitamin K Dependent Bone Protein) OR (Protein, Bone Gla) OR (4 Carboxyglutamic Protein, Bone) OR (Vitamin K Dependent Bone Protein)) |  |  |  |
|  |  | #2 | TI= ((collagen type I trimeric cross linked peptide) OR (Bone gamma Carboxyglutamic Acid Protein) OR (trimeric cross linked peptide collagen type I) OR (C terminal type I collagen telopeptide) OR (CTCLP) OR (CTx telopeptide) OR (serum carboxyterminal telopeptide type I collagen) OR (ICTP peptide) OR (C terminal telopeptide of type I collagen) OR (COOH terminal telopeptide of type I collagen) OR (C terminal cross linking telopeptide, collagen type I) OR (N telopeptide) OR (N terminal type I collagen telopeptide) OR (NTx telopeptide) OR (pyridinoline cross linked carboxy terminal telopeptide, collagen type I) OR (C telopeptide) OR (i ICTP) OR (CTX)) OR AK= ((collagen type I trimeric cross linked peptide) OR (Bone gamma Carboxyglutamic Acid Protein) OR (trimeric cross linked peptide collagen type I) OR (C terminal type I collagen telopeptide) OR (CTCLP) OR (CTx telopeptide) OR (serum carboxyterminal telopeptide type I collagen) OR (ICTP peptide) OR (C terminal telopeptide of type I collagen) OR (COOH terminal telopeptide of type I collagen) OR (C terminal cross linking telopeptide, collagen type I) OR (N telopeptide) OR (N terminal type I collagen telopeptide) OR (NTx telopeptide) OR (pyridinoline cross linked carboxy terminal telopeptide, collagen type I) OR (C telopeptide) OR (i ICTP) OR (CTX)) OR AB= ((collagen type I trimeric cross linked peptide) OR (Bone gamma Carboxyglutamic Acid Protein) OR (trimeric cross linked peptide collagen type I) OR (C terminal type I collagen telopeptide) OR (CTCLP) OR (CTx telopeptide) OR (serum carboxyterminal telopeptide type I collagen) OR (ICTP peptide) OR (C terminal telopeptide of type I collagen) OR (COOH terminal telopeptide of type I collagen) OR (C terminal cross linking telopeptide, collagen type I) OR (N telopeptide) OR (N terminal type I collagen telopeptide) OR (NTx telopeptide) OR (pyridinoline cross linked carboxy terminal telopeptide, |  |  |  |

|  | \|  \|  \| collagen type I) OR (C telopeptide) OR (i ICTP) OR (CTX)) \| 29997  51352  63131  122 \| \| --- \| --- \| --- \| --- \| \| #3 \| TI=((procollagen Type I N terminal peptide) OR (P I P peptide) OR (Type I procollagen N terminal peptide) OR (P1NP peptide) OR (amino terminal propeptide of type I collagen) OR (PINP peptide) OR (fetal antigen 2, human) OR (foetal antigen 2, human) OR (FA2 antigen, human) OR (aminoterminal propeptide of type I collagen, human) OR (collagen type I, amino propeptide, human) OR (PINP)) OR AB=((procollagen Type I N terminal peptide) OR (P I P peptide) OR (Type I procollagen N terminal peptide) OR (P1NP peptide) OR (amino terminal propeptide of type I collagen) OR (PINP peptide) OR (fetal antigen 2, human) OR (foetal antigen 2, human) OR (FA2 antigen, human) OR (aminoterminal propeptide of type I collagen, human) OR (collagen type I, amino propeptide, human) OR (PINP)) OR AK=((procollagen Type I N terminal peptide) OR (P I P peptide) OR (Type I procollagen N terminal peptide) OR (P1NP peptide) OR (amino terminal propeptide of type I collagen) OR (PINP peptide) OR (fetal antigen 2, human) OR (foetal antigen 2, human) OR (FA2 antigen, human) OR (aminoterminal propeptide of type I collagen, human) OR (collagen type I, amino propeptide, human) OR (PINP)) \| \| #4 \| TI=((NAFLD) OR (Non alcoholic Fatty Liver Disease) OR (Nonalcoholic Fatty Liver Disease) OR (Fatty Liver, Nonalcoholic) OR (Fatty Livers, Nonalcoholic) OR (Liver, Nonalcoholic Fatty) OR (Livers, Nonalcoholic Fatty) OR (Nonalcoholic Fatty Liver) OR (Nonalcoholic Fatty Livers) OR (Nonalcoholic Steatohepatitis) OR (Nonalcoholic Steatohepatitides) OR (Steatohepatitides, Nonalcoholic OR hepato steatosis) OR (non alcoholic hepatosteatosis) OR (non alcoholic liver steatosis) OR (non alcoholic steatotic hepatopathy) OR (non alcoholic fatty liver) OR (non alcoholic fatty liver disease) OR (non alcoholic FLD) OR (non  alcoholic hepatic steatosis) OR (nonalcoholic FLD) OR (nonalcoholic hepatosteatosis) OR (nonalcoholic hepatic steatosis) OR (nonalcoholic liver steatosis) OR (Steatohepatitis, Nonalcoholic) OR (Steatohepatitides, Nonalcoholic) OR (Nonalcoholic Steatohepatitis) OR (Nonalcoholic Steatohepatitides) OR (Fatty Livers, Nonalcoholic) OR (Liver, Nonalcoholic Fatty) OR (Nonalcoholic Fatty Livers) OR (Livers, Nonalcoholic Fatty) OR (NAFLD) OR (Non alcoholic Fatty Liver Disease) OR (Nonalcoholic Fatty Liver) OR (Nonalcoholic Fatty Liver Disease) OR (Fatty Liver, Nonalcoholic)) OR AB=((NAFLD) OR (Non alcoholic Fatty Liver Disease) OR (Nonalcoholic Fatty Liver Disease) OR (Fatty Liver, Nonalcoholic) OR (Fatty Livers, Nonalcoholic) OR (Liver, Nonalcoholic Fatty) OR (Livers, Nonalcoholic Fatty) OR (Nonalcoholic Fatty Liver) OR (Nonalcoholic Fatty Livers) OR (Nonalcoholic Steatohepatitis) OR (Nonalcoholic Steatohepatitides) OR (Steatohepatitides, Nonalcoholic OR hepato steatosis) OR (non alcoholic hepatosteatosis) OR (non alcoholic liver steatosis) OR (non alcoholic steatotic hepatopathy) OR (non alcoholic fatty liver) OR (non alcoholic fatty liver disease) OR (non alcoholic FLD) OR (non alcoholic hepatic steatosis) OR (nonalcoholic FLD) OR (nonalcoholic hepatosteatosis) OR (nonalcoholic hepatic steatosis) OR (nonalcoholic liver steatosis) OR (Steatohepatitis, Nonalcoholic) OR (Steatohepatitides, Nonalcoholic) OR (Nonalcoholic Steatohepatitis) OR (Nonalcoholic Steatohepatitides) OR (Fatty Livers, Nonalcoholic) OR (Liver, Nonalcoholic Fatty) OR (Nonalcoholic Fatty Livers) OR (Livers, Nonalcoholic Fatty) OR (NAFLD) OR (Non alcoholic Fatty Liver Disease) OR (Nonalcoholic Fatty Liver) OR (Nonalcoholic Fatty Liver Disease) OR (Fatty Liver, Nonalcoholic)) OR AK=((NAFLD) OR (Non alcoholic Fatty Liver Disease) OR (Nonalcoholic Fatty Liver Disease) OR (Fatty Liver, Nonalcoholic) OR (Fatty Livers, Nonalcoholic) OR (Liver, Nonalcoholic Fatty) OR (Livers, Nonalcoholic Fatty) OR (Nonalcoholic Fatty Liver) OR (Nonalcoholic Fatty Livers) OR (Nonalcoholic Steatohepatitis) OR (Nonalcoholic Steatohepatitides) OR (Steatohepatitides, Nonalcoholic OR hepato steatosis) OR (non alcoholic hepatosteatosis) OR (non alcoholic liver steatosis) OR (non alcoholic steatotic  hepatopathy) OR (non alcoholic fatty liver) OR (non alcoholic fatty liver disease) OR (non alcoholic FLD) OR (non alcoholic hepatic steatosis) OR (nonalcoholic FLD) OR (nonalcoholic hepatosteatosis) OR (nonalcoholic hepatic steatosis) OR (nonalcoholic liver steatosis) OR (Steatohepatitis, Nonalcoholic) OR (Steatohepatitides, Nonalcoholic) OR (Nonalcoholic Steatohepatitis) OR (Nonalcoholic Steatohepatitides) OR (Fatty Livers, Nonalcoholic) OR (Liver, Nonalcoholic Fatty) OR (Nonalcoholic Fatty Livers) OR (Livers, Nonalcoholic Fatty) OR (NAFLD) OR (Non alcoholic Fatty Liver Disease) OR (Nonalcoholic Fatty Liver) OR (Nonalcoholic Fatty Liver Disease) OR (Fatty Liver, Nonalcoholic)) \| \| #5 \| TI=((NASH) OR (nonalcoholic steatohepatitis) OR (non alcohol steato hepatitis) OR (non alcohol steatohepatitis) OR (non alcoholic steato hepatitis) OR (non alcohol steato hepatitis) OR (non alcohol steatohepatitis) OR (non alcoholic steatohepatitis) OR (non alcoholic steatosis hepatitis) OR (non alcoholic steatotic hepatitis) OR (nonalcohol steato hepatitis) OR (nonalcohol steatohepatitis) OR (nonalcoholic fatty liver inflammation) OR (nonalcoholic steato hepatitis) OR (nonalcoholic steatosis hepatitis) OR (nonalcoholic steatotic hepatitis)) OR AB=((NASH) OR (nonalcoholic steatohepatitis) OR (non alcohol steato hepatitis) OR (non alcohol steatohepatitis) OR (non alcoholic steato hepatitis) OR (non alcohol steato hepatitis) OR (non alcohol steatohepatitis) OR (non alcoholic steatohepatitis) OR (non alcoholic steatosis hepatitis) OR (non alcoholic steatotic hepatitis) OR (nonalcohol steato hepatitis) OR (nonalcohol steatohepatitis) OR (nonalcoholic fatty liver inflammation) OR (nonalcoholic steato hepatitis) OR (nonalcoholic steatosis hepatitis) OR (nonalcoholic steatotic hepatitis)) OR AK=((NASH) OR (nonalcoholic steatohepatitis) OR (non alcohol steato hepatitis) OR (non alcohol steatohepatitis) OR (non alcoholic steato hepatitis) OR (non alcohol steato hepatitis) OR (non alcohol steatohepatitis) OR (non alcoholic steatohepatitis) OR (non alcoholic steatosis hepatitis) OR (non alcoholic steatotic hepatitis) OR (nonalcohol steato hepatitis) OR (nonalcohol steatohepatitis) OR (nonalcoholic fatty liver inflammation) OR (nonalcoholic steato hepatitis) OR (nonalcoholic steatosis hepatitis) OR (nonalcoholic steatotic hepatitis)) \| \| #6 \| (#5 or #4) and (#1 or #2 or #3) \| \| CNKI \| TKA=('NAFLD' + '非酒精性脂肪肝' + 'NASH' + '非酒精性肝硬化') * ('骨转换' + '骨钙素' + 'CTX' + 'PINP' + '骨代谢') \| \| 57 \|   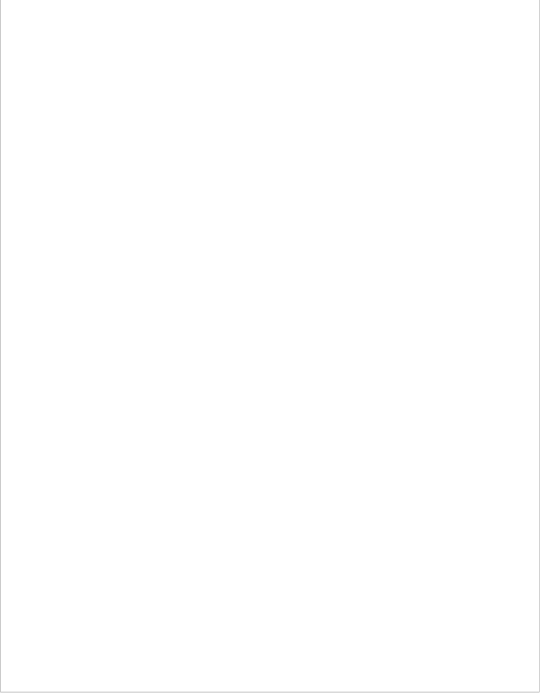 |
| --- | --- | --- | --- | --- | --- | --- | --- | --- | --- | --- | --- | --- | --- | --- | --- | --- | --- |

**Supplementary Table S1：**Data extraction of included studies in the meta-analysis

| Author | Year | Region | Population | Age(mean, SD) | Gender(female, male) |
| --- | --- | --- | --- | --- | --- |
| Qi Huang | 2021 | China | 90 | 38.86±6.83 | 46,44 |
| Yulei Qu | 2021 | China | 360 | 55.65±9.13 | 191,169 |
| Li Zhang | 2015 | China | 800 | 54.10±4.47 | 0,800 |
| Xin Liao | 2019 | China | 88 | 40.88±5.54 | 38,50 |
| Jing Du | 2015 | China | 174 | 66.89±10.18 | 123,51 |
| Ran Cui | 2013 | China | 224 | 59.55±5.73 | 99,125 |
| Xiaolei Hu | 2016 | China | 368 | 40.11±10.18 | 273,95 |
| JunJie Liu | 2013 | China | 1683 | 37.52±11.23 | 1683,0 |
| Jianxin Dou | 2013 | China | 1158 | 54.02±8.67 | 1158,0 |
| Yu-qi LUO | 2015 | China | 733 | 56.24±4.51 | 0,733 |
| H. DENG | 2018 | China | 540 | 50.42±5.05 | 540,0 |
| M. Gudowska-Sawczuk | 2017 | Poland | 58 | 26-88 | 30,28 |
| Dong-Yun Lee | 2018 | Korea | 3737 | 54.48±2.50 | 0,3737 |
| Hae Jin Yang | 2015 | Korea | 859 | 45.00±6.72 | 859,0 |
| Dalal Al-Akabi | 2021 | *Iraq* | 800 | 30-50 | 800,0 |
| YUSUF YILMAZ | 2011 | Turkey | 174 | 48±7.56 | 87,87 |
| Stergios A. Polyzos | 2015 | Japan | 47 | 53.03±2.46 | 34,13 |
| Henrik Maagensen | 2018 | *Denmark* | 17 | 52.97±16.93 | 17,0 |
